# Cell Culture Characterization of Prooxidative Chain-Transfer Agents as Novel Cytostatic Drugs

**DOI:** 10.3390/molecules26216743

**Published:** 2021-11-08

**Authors:** Victoria Heymans, Sascha Kunath, Parvana Hajieva, Bernd Moosmann

**Affiliations:** 1 Evolutionary Biochemistry and Redox Medicine, Institute for Pathobiochemistry, University Medical Center of the Johannes Gutenberg University, 55128 Mainz, Germany; vichey2405@gmail.com (V.H.); sakunath@uni-mainz.de (S.K.); 2 Institute for Translational Medicine, MSH Medical School Hamburg, 20457 Hamburg, Germany; parvana.hajieva@medicalschool-hamburg.de

**Keywords:** chain-transfer agent, chemotherapy, free radical chain reaction, lipid peroxidation, lipophilic thiol, oxidative cell death, prooxidative drug, radical propagation, rate-limiting step

## Abstract

Prooxidative therapy is a well-established concept in infectiology and parasitology, in which prooxidative drugs like artemisinin and metronidazole play a pivotal clinical role. Theoretical considerations and earlier studies have indicated that prooxidative therapy might also represent a promising strategy in oncology. Here, we have investigated a novel class of prooxidative drugs, namely chain-transfer agents, as cytostatic agents in a series of human tumor cell lines in vitro. We have found that different chain-transfer agents of the lipophilic thiol class (like dodecane-1-thiol) elicited half-maximal effective concentrations in the low micromolar range in SY5Y cells (human neuroblastoma), Hela cells (human cervical carcinoma), HEK293 cells (immortalized human kidney), MCF7 cells (human breast carcinoma), and C2C12 cells (mouse myoblast). In contrast, HepG2 cells (human hepatocellular carcinoma) were resistant to toxicity, presumably through their high detoxification capacity for thiol groups. Cytotoxicity was undiminished by hypoxic culture conditions, but substantially lowered after cellular differentiation. Compared to four disparate, clinically used reference compounds in vitro (doxorubicin, actinomycin D, 5-fluorouracil, and hydroxyurea), chain-transfer agents emerged as comparably potent on a molar basis and on a maximum-effect basis. Our results indicate that chain-transfer agents possess a promising baseline profile as cytostatic drugs and should be explored further for anti-tumor chemotherapy.

## 1. Introduction

Despite tremendous successes in the last few decades, there is a continuing demand for new lead structures in oncology. One of the reasons behind this demand is the still sobering survival rate observed with many different types of cancer. For instance, 5-year-survival rates after cancer diagnosis in the US between 2008 and 2014 have been reported to be only 9% for pancreas, 18% for liver, 19% for esophagus, and 19% for lung [1]. Moreover, the increasing cost of many newer drugs has become a serious concern [2]. To meet these challenges, drug candidates would be particularly interesting that reach beyond the established therapeutic principles [3]. In general, the most difficult task in generating novel and tolerable cytostatic drugs for chemotherapy has been the identification of new biochemical aspects in which tumor cells are substantially and “drugably” different from normal, differentiated cells and normal, but regularly dividing cells such as stem cells.

In recent years, there has been an increasing awareness that redox metabolism in tumor cells is substantially altered, pointing at the presence of a generalized prooxidant state [4,5,6,7]. Specifically, certain tumor cells appear to exhibit reduced antioxidant enzyme activities [8] and increased production of reactive oxidative species (ROS) due to flavoprotein activation [9,10] or, potentially, mutation accumulation in the mitochondrial DNA [5]. In consequence, a prooxidative treatment strategy for cancer was proposed, based on the idea that an additional elevation of ROS levels in cancer cells would lift these cells above a toxic threshold, whereas the same lift in normal cells would perhaps damage, but not kill the cells [4,6,7]. The latter idea was rationalized by the recognition that established therapeutic regimes such as radiotherapy [11,12] or photodynamic therapy [13] also have a strong prooxidant functional component.

Despite an impressive number of different approaches towards prooxidant tumor therapy [4,6,14,15,16], none of those has seemingly involved the direct “sensing” of the elevated level of ROS or free radicals in tumor cells as criterion to distinguish between tumor cells and normal cells. Therefore, based on recent work describing the unique catalytic behavior of so-called “chain-transfer agents” in biological cells [17], we have investigated the cytostatic potential of these prooxidative agents in a series widely utilized tumor cell lines in vitro. Chain-transfer agents are generally reducing chemicals, whose prooxidative action in vivo only materializes after intracellular oxidation by endogenous free radicals. Thus, we hypothesized that these agents may indeed represent sensors of the elevated free radical tone in tumor cells.

Very different structural classes of compounds can exhibit chain-transfer activity in the test tube, among them metal complexes [18], thiols [19], trithiocarbonates [20] and nitroxides [21]. All of these compounds are widely used in polymer chemistry to control the outcome of radical polymerization processes [22]. In the present tumor biological study, we have focused on lipophilic thiols as lead compounds for three main reasons: (i) they appeared to be the most compatible with an aqueous, biological context, (ii) they have already been demonstrated to exhibit chain-transfer activity in cell culture and in vivo [17], and (iii) there is strong evidence that thiol-type chain-transfer activity might have significantly shaped biochemical evolution in the past [23,24].

## 2. Results

### 2.1. Comparative Evaluation of Chain-Transfer Agents as Anti-Proliferative Drugs in Four Human Tumor Cells Lines

A series of linear primary thiols with incremental lipophilicity, ranging from octane-1-thiol (8SH) to octadecane-1-thiol (18SH) (Table 1), was investigated in cell culture for potential cytostatic effects at nanomolar and micromolar concentrations during a 3-day incubation period.

**Figure 1:**
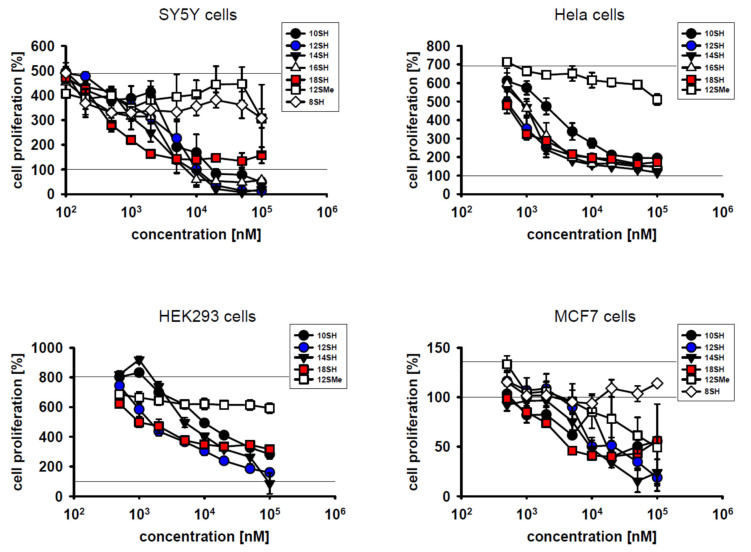
Inhibitory effect of different thiol-type chain-transfer agents on cellular proliferation in SY5Y cells, Hela cells, HEK293 cells and MCF7 cells. Compound abbreviations are explained in Table 1. Cellular proliferation was assessed by metabolic MTT assay as described in the Materials and Methods. The control line at 100% represents the metabolic activity of the adherent cells at the beginning of the experiment; the variable, upper control line represents the final activity of the cells after the 3-day experiment. Note that MCF7 cells exhibited a much lower cell division rate than the other cells, amounting to less than one population doubling over the course of the experiment.

The results in Figure 1 indicate that lipophilic thiols were efficient inhibitors of cell proliferation in diverse types of cultivated tumor cells, namely SY5Y human neuroblastoma cells, Hela human cervical carcinoma cells, HEK293 immortalized human kidney cells, and MCF7 human breast carcinoma cells. With respect to the inhibition of cell proliferation, half-maximal effective concentrations (EC_50_ values) in the single-digit micromolar range were attained in all cell lines (Figure 1; Table 2). In general, more lipophilic compounds were more effective in terms of their EC_50_ values. This relationship was not linear, however, as a strong increase in efficacy was noted between 8SH and 10SH, whereas only a modest additional increase was seen with the more highly lipophilic compounds. Hence, cytotoxicity was apparently restricted to compounds beyond a certain lipophilicity threshold (logP = 4) as noted before [17]. This observation probably relates to the fact that less lipophilic thiols, after conversion to chain-transferring thiyl radicals, might reversibly protrude from the lipid bilayer and react with glutathione, which would blunt chain-transfer catalysis [17]. More highly lipophilic thiyl radicals, however, are probably restricted to the lipid bilayer permanently and thus cannot be scavenged by aqueous glutathione.

On the other hand, very long-chain thiols like 18SH tended to be somewhat less effective in the killing of already present cells, potentially due to limited penetration of established cells in the culture. Still, such a cytotoxic effect (i.e., a value of less than 100% in the graphs in Figure 1) was only observed in certain cell lines like SY5Y, but not in Hela cells. Notably, the compound 12SMe, which is not a chain-transfer agent, but a chain-transfer negative control for the compound 12SH, generally did not affect cell proliferation up to the highest concentration tested (100 µM) (except in MCF7 cells; Table 2). This result verifies that the thiol group of the active agents caused their toxicity, as would be expected for chain-transfer agents [17,19]. Nonspecific alkyl group overload effects were apparently irrelevant for the noted cytostatic effects.

### 2.2. Effect of Cellular Differentiation on Chain-Transfer Agent Cytotoxicity

**Figure 2:**
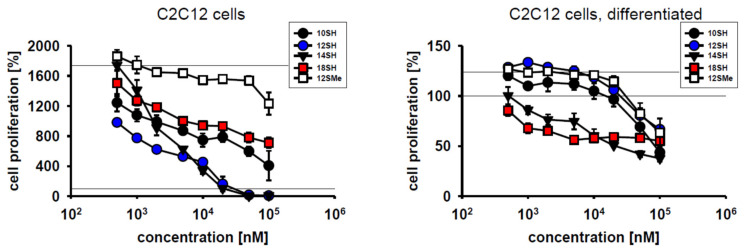
Cytotoxic effect of chain-transfer agents in naïve vs. differentiated C2C12 cells. Compound designations are used as in Table 1. The employed differentiation protocol involving serum withdrawal of a confluent culture led to a significant reduction of proliferation from ~1700% to ~125% as assessed by MTT assay.

Cytotoxic compounds for clinical use should exhibit efficacy towards dividing cells, but should ideally spare differentiated, quiescent cells. To test the behavior of chain-transfer agents in this respect, mouse myoblast C2C12 cells were chosen because they divide very rapidly under cultivation conditions with FCS, but differentiate rapidly upon serum withdrawal at high cell densities [25]. Within 3 days of cultivation, C2C12 cells achieved approximately 4 population doublings (~1700% proliferation) (Figure 2). C2C12 cell proliferation was not inhibited by the thioether control compound 12SMe, whereas the thiol compound 12SH fully blocked cell division at a concentration of 20 µM, with half-maximal efficacy at approximately 1 µM (Figure 2). Differentiated C2C12 cells were significantly less affected by chain-transfer agent toxicity, as the obtained survival curves were shifted to the right by about one order of magnitude. This indicates an approximately 10x lower toxicity of chain-transfer agents upon cellular differentiation (Figure 2, Table 2). The highly lipophilic alkyl thiol 18SH apparently reduced the viability of the plated, differentiated cells by up to 50%, but without a clear dose-response. This finding might indicate some nonspecific toxicity of long-chain alkyl compounds in differentiated myoblasts that is unrelated to chain-transfer activity. The latter idea is supported by the fact that in differentiated cells, the formerly observed, wide gap between 12SH and 12SMe (Figure 2, left) completely collapsed, with coinciding survival curves for both compounds (Figure 2, right).

### 2.3. Potential Limitations of Chain-Transfer Agents as Cytostatic Drugs

**Figure 3:**
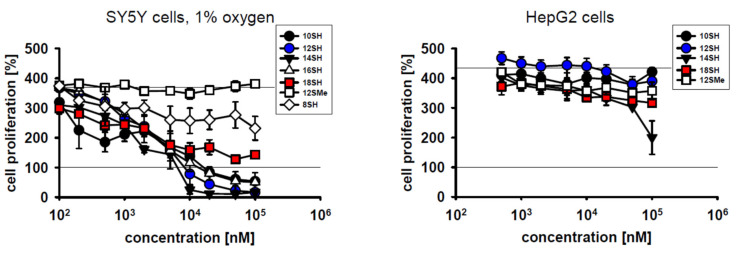
Cytotoxicity of chain-transfer agents in SY5Y cells under hypoxic culture conditions and in HepG2 cells. Compound-treated SY5Y cells were cultivated at 1% oxygen partial pressure under otherwise unchanged conditions for 3 days. Hypoxic culture conditions only modestly lowered baseline proliferation of the SY5Y cells from ~500% to ~400% as per MTT assay. HepG2 hepatocellular carcinoma cells were cultivated at 20% oxygen partial pressure and evaluated as in Figure 1.

Low tumor oxygenation (hypoxia) in solid tumors is of major relevance for tumor cell behavior and treatability [26]. Specifically, tumor hypoxia may induce genomic instability of the tumor cells, exert local immunosuppressive effects, and it frequently leads to cancer cell spreading and tumor dissemination [26,27,28]. Importantly, hypoxia is known to limit the efficacy of radiotherapy [26]. Hence, it was investigated whether hypoxic conditions may also curtail the cytostatic potency of prooxidative chain-transfer agents. Cultivation of SY5Y cells under 1% oxygen slightly reduced their baseline proliferative capacity as expected (Figure 3). However, there were no relevant changes in the cytostatic and cytotoxic activity of the tested compounds (Figure 1 and Figure 3); EC_50_ values were essentially identical at 1% O_2_ and 20% O_2_ (Table 2). This somewhat surprising result may be accounted for by the fact that even at only 1% O_2_, other steps of prototypical radical chain reactions are slower (and thus rate-limiting) than steps involving the O_2_ molecule itself, as detailed in the Discussion.

Hepatocellular carcinoma is a malignant disease characterized by low 5-year survival rates of about 15% [29]. One of the origins of therapeutic futility in this cancer is cellular chemoresistance involving very effective drug expulsion and drug metabolism, among other mechanisms [29]. Human hepatocellular carcinoma cells (HepG2 cells) were thus added to the spectrum of tumor cell lines investigated in this work. The results in Figure 3 demonstrate that HepG2 cells were indeed entirely resistant to lipophilic thiol toxicity, as plausibly explained by the superior thiol detoxification capacity described for the liver [30]. Whether chain-transfer agents with other lead structures may overcome HepG2 cell resistance remains to be determined.

### 2.4. Comparison of 12SH and 18SH with Four Clinically Established Cytostatic Drugs

**Figure 4:**
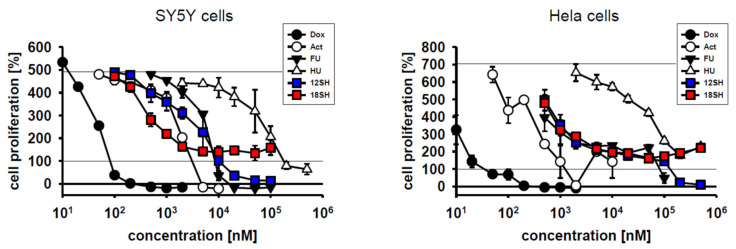
Cytostatic efficacy of chain-transfer agents in comparison with doxorubicin (Dox), actinomycin D (Act), 5-fluorouracil (FU) and hydroxyurea (HU). SY5Y cells and Hela cells were investigated after 3-day treatment under standard cultivation conditions as in Figure 1; the curves for 12SH and 18SH were adopted from that figure.

To achieve a quantitative assessment of chain-transfer agent cytostatic potential in direct comparison with established anti-tumor drugs, the compounds doxorubicin (a DNA intercalator and topoisomerase inhibitor), actinomycin D (a transcriptional inhibitor), 5-fluorouracil (a thymidylate synthase inhibitor), and hydroxyurea (a ribonucleotide reductase inhibitor) were chosen as reference standards. These compounds were investigated in SY5Y cells and Hela cells under identical conditions as the chain-transfer agents before. The results in Figure 4 demonstrate that all four clinical reference compounds acted as cytostatic drugs in both cell lines, but with vastly differing molar efficacies spanning five orders of magnitude; EC_50_ values are provided in Table 2. Notably, the chain-transfer agents 12SH and 18SH were both localized right in the middle of the efficacy spectrum, most closely resembling actinomycin D in SY5Y cells, and 5-fluorouracil in Hela cells. Certain reference compounds, namely doxorubicin and actinomycin D, were particularly effective in the killing of the initially plated cells (i.e., they achieved a value of less than 100% in the graphs in Figure 4), beyond their inhibition of cell proliferation. Hydroxyurea, however, similarly as 18SH, only inhibited proliferation, but did not kill existing cells up to the highest concentration tested (500 µM). It is unclear at present whether the killing of initially plated cells under the employed conditions should be viewed as desirable for an anti-tumor drug, because it might also predict the killing of non-tumor, preexisting cells in vivo.

## 3. Discussion

In this work, we provide initial evidence that chain-transfer agents might become useful anti-cancer drugs of an entirely novel mechanistic class, for which we would propose the term “prooxidative amplifiers”. With EC_50_ values in the low micromolar range, chain-transfer agents exerted cytostatic effects at approximately the same concentrations as traditional and clinically administered anti-cancer agents like actinomycin D and fluorouracil under identical testing conditions (Figure 4, Table 2). The cytostatic activity of the chain-transfer agents was undiminished by hypoxic culture conditions (Figure 3), which is relevant for the potential treatment of solid, hypoxic tumors. Cellular differentiation, however, led to an increase in EC_50_ in the investigated cell line (C2C12) by approximately one order of magnitude, and it was accompanied by substantially lowered maximum effects (Figure 2), which would fulfill a second, important prerequisite for anti-tumor drugs. On the other hand, the chain-transfer agents were ineffective in hepatocellular carcinoma cells (Figure 3), presumably due to rapid drug metabolism and inactivation. Therefore, chain-transfer agents are obviously not universal cytotoxins, but will require serial screening for the most promising fields of application.

In a cell biological context, chain-transfer agents of the lipophilic thiol class accelerate free radical chain reactions, which leads to a heightened toxicity of the low levels of free radicals naturally produced by the cell [17]. In particular, chain-transfer agents in normal human diploid fibroblasts expedited lipid peroxidation, as evidenced by lowered levels of phospholipid poly-unsaturated fatty acids (PUFAs), and sharply elevated the levels of 8-isoprostanes and trans-fatty acids [17]. Moreover, increased protein oxidation, especially of membrane proteins, was observed, which was accompanied by a massively induced cellular stress response. Similar findings were made in *C. elegans* in vivo [17]. Hence, chain-transfer agents in living cells evoke a well-characterized spectrum of biochemical changes and subsequent compensatory responses related to oxidative stress. Essential starting point of this prooxidative amplification is the presence of naturally formed, endogenous initiator radicals, because in contrast to many classic prooxidant pharmaceuticals like artemisinin, chain-transfer agents by themselves are reducing chemicals whose complete catalytic cycle has to be considered in order to appreciate their overall prooxidant activity, as detailed below.

As many tumor cells appear to possess higher steady-state levels of endogenous initiator radicals than normal cells [4,6,8,9,10], the accelerating catalysis of the chain-transfer agents might be used to achieve a specific toxic effect in tumor cells, whereas normal cells would be relatively spared [4,6,7]. Unlike other prooxidant anti-cancer strategies evaluated so far [4,6], chain-transfer agents would thus mechanistically respond to the difference in oxidant tone between tumor cells and normal cells, to proportionally potentiate this difference [17]. Thereby, they would act as “pathologically activated therapeutics” [31]. In our view, this mechanistic feature might be an important advantage compared to more traditional strategies such as antioxidant enzyme inhibition or direct prooxidation [4,6], which usually add oxidative reactivity to many cell types in a relatively non-specific fashion.

**Figure 5:**
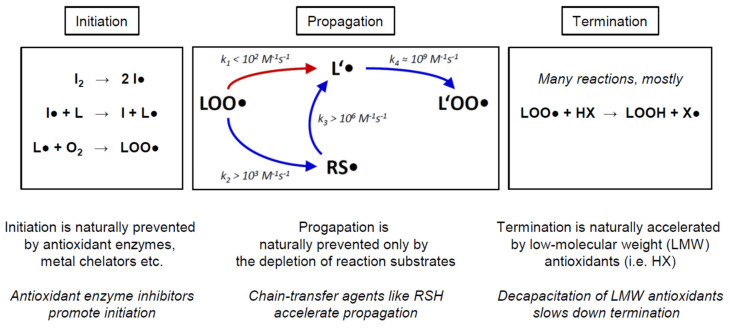
Prooxidative mechanism of chain-transfer agents in living cells, exemplified by the lipid peroxidation reaction. Chemical reactions involving free radicals in living cells frequently present as radical chain reactions (RCRs). RCR possess three kinetically independent elementary steps, namely initiation, propagation, and termination. Antioxidant or prooxidant chemicals and enzymes are generally characterized by their specific interference with only one of these elementary steps. For example, hydrogen peroxide typically accelerates initiation, whereas vitamin E accelerates termination; both do not affect propagation. In contrast, chain transfer agents specifically accelerate radical propagation. More details are provided in the Discussion. The abbreviations denote: initiator (I_2_); initiator radical (I●); lipid (L); lipid radical (L●); molecular oxygen (O_2_); lipid peroxyl radical (LOO●); a second lipid (L’, omitted for clarity); a second lipid radical (L’●); a second lipid peroxyl radical (L’OO●); lipophilic thiol (RSH, omitted for clarity); lipophilic thiol radical (RS●); low-molecular weight antioxidant (HX); lipid hydroperoxide (LOOH); antioxidant radical (X●); rate constant (k_X_). The propagation scheme and the rate constants were adopted from [17].

In the following, we would like to provide a brief overview of the biochemical mechanism of chain-transfer agents in vivo, to illustrate the differences between the various prooxidative strategies proposed for cancer treatment. Biological and cytotoxic damage from free radicals is foremost related to radical chain reactions, which can produce extensive damage once started (Figure 5). The arguably most important such chain reaction is lipid peroxidation [32,33]. As sketched in Figure 5, lipid peroxidation is started by the initiation step, which involves the attack of a variable initiator radical (I●) on a lipid (L), usually followed by a rapid reaction of the ensuing lipid radical (L●) with ambient molecular oxygen (O_2_) to yield a lipid peroxyl radical (LOO●). During propagation, the lipid peroxyl radical (LOO●) slowly radicalizes another lipid (L’) to yield another lipid radical (L’●), which again rapidly adds oxygen to produce a lipid peroxyl radical (L’OO●). The latter product may then attack yet another lipid, resulting in a potentially endless chain reaction as long as enough substrates (lipid L’ and O_2_) are present. Termination may be effectuated by a variety of mechanisms, predominantly the donation of a hydrogen radical by a low-molecular weight antioxidant (HX) to a lipid peroxyl radical (L’OO●). This step results in two relatively stable products to be disposed of or recycled, namely a lipid hydroperoxide (LOOH) and an antioxidant radical (X●).

The rate-limiting step of propagation is the formation of the lipid radical L’● (Figure 5, red arrow). Importantly, it has been argued that propagation may also constitute the rate-limiting step of the overall chain reaction in many biological systems, since it is certainly the most difficult of the three elementary steps (initiation, propagation, termination) to be modified by acute cellular intervention or long-term evolutionary adaptation [34,35]. Notably, it is this very step that is bypassed and thereby accelerated by chain-transfer agents such as lipophilic thiols (RSH). Moreover, adverse chain-transfer catalysis by lipid bilayer thiol groups may also explain why these groups appear to be negatively selected for during evolution [24,36]. Representative rate constants for the propagation reaction are given in Figure 5, indicating that thiol-type chain transfer agents would usually accelerate propagation by more than 10×, yet depending on the actual substrate concentrations present. Detailed quantitative considerations analyzing these factors and their biological implications have been published [17].

Importantly, propagation cannot be easily modified by adaptive enzymatic responses of the cell, as it formally depends only on the concentration of the lipid substrate L’, the concentration of oxygen, and the temperature. In a tumor biological context, hardly any of these factors may become relevant as a mechanism of tumor cell chemoresistance. First, temperature is widely constant in the human body. Second, oxygen concentration is certainly of interest and has been extensively discussed in terms of its impact on tumor behavior, progression, and treatability [5,26,27,28]. However, as regards its impact on lipid peroxidation and other radical chain reactions, the reaction rate of carbon-centered radicals with oxygen is so fast (Figure 5, k_4_ ≈ 10^9^ M^−1^s^−1^) that even a 100× lower oxygen concentration in tumors arguably would not make this reaction rate-relevant [17]. Experimentally, we have investigated SY5Y cells cultivated under 20% and 1% oxygen partial pressure, and we have not seen any notable differences in their susceptibility to chain-transfer agent toxicity (Figure 3, Table 2). Finally, the concentrations of the lipid substrates need to be considered. As judged from the reactivities of saturated vs. mono-unsaturated vs. poly-unsaturated fatty acids, only the latter are of general relevance [17]. Because the degree and type of lipid unsaturation are largely preset by the biological species and the tissue that is analyzed [37,38], however, there is only a modest chance for a tumor cell to adaptively respond to and thus escape the toxic action of a chain-transfer agent. Altered PUFA usage has been described for a variety of tumor cell types already, but the effect sizes were generally smaller than 2× and thus negligible in a reaction rate context [39,40,41]. Therefore, an adaptive escape of tumor cells from chain-transfer agent toxicity is very unlikely, such that it appears paramount to assess and identify those tumor cell types whose baseline properties at the outset are the most promising [8,9,10].

To date, there is only basic information available about the pharmacodynamics and toxicology of the employed chain-transfer agents. According to the manufacturer-provided chemical safety record, the reference compound 12SH is non-genotoxic (as per Ames test, micronucleus test and sister chromatid exchange assay), non-teratogenic, and devoid of reproductive toxicity in mice [42]. After oral application in rats, the half-lethal dose (LD_50_) was higher than 5000 mg/kg (~25 mmol/kg), apparently the highest dose tested. For comparison, 5-fluorouracil was positive in all genotoxicity assays and half-lethal in rats at 230 mg/kg (~1.8 mmol/kg) [43]. Actinomycin D, in turn, has been reported to be half-lethal in rats already at 7.2 mg/kg (~0.0057 mmol/kg) following oral administration [44].

The current study has two major limitations. First, in vivo data from an accepted animal model are not available yet. Such data would be essential for the assessment of the selectivity of the presented chain-transfer agents for tumor cells vs. normal cells in a whole-body context. Second, the chemical mechanism of chain-transfer catalysis elaborated before in fibroblasts [17] was not rechecked in the presently investigated tumor cells. Still, since the original report [17] has shown coherent effects in two rather different experimental systems (diploid human lung fibroblasts and *C. elegans* nematodes), it appears plausible that a related mechanism also accounts for the here described cytotoxicity in tumor cells.

## 4. Materials and Methods

### 4.1. Chemicals and Reagents

The investigational thiols and thioethers were obtained from the following sources: octane-1-thiol (8SH; CAS 111-88-6) was from Sigma-Aldrich, St. Louis, MO, USA (#471836, purity ≥98.5%); decane-1-thiol (10SH; CAS 143-10-2) was from Sigma-Aldrich (#705233, purity 99%); dodecane-1-thiol (12SH; CAS 112-55-0) was from Sigma-Aldrich (#471364, purity ≥98%); tetradecane-1-thiol (14SH; CAS 2079-95-0) was from Sigma-Aldrich (#87193, purity ≥98%); hexadecane-1-thiol (16SH; CAS 2917-26-2) was from Alfa Aesar, Ward Hill, MA, USA (#L15099, purity 97%); octadecane-1-thiol (18SH; CAS 2885-00-9) was from Sigma-Aldrich (#O1858, purity 98%); 1-methylsulfanyldodecane (12SMe; CAS 3698-89-3) was from Sigma-Aldrich (#641480, purity 97%). The lipophilicities of these compounds were calculated as octanol-water partition coefficients (logP) with the ChemPropPro tool that is part of the ChemBio3D 13.0 software package (PerkinElmer, Waltham, MA, USA).

Reference cytostatic drugs were purchased from the following suppliers: doxorubicin hydrochloride (Dox; CAS 25316-40-9) was from Cayman Chemicals, Ann Arbor, MI, USA (#15007; purity ≥98%); actinomycin D (Act; CAS 50-76-0) was from Cayman Chemicals (#11421; purity ≥95%); 5-fluorouracil (FU; CAS 51-21-8) was from Sigma-Aldrich (#F6627; purity ≥99%); hydroxyurea (HU; CAS 127-07-1) was from Sigma-Aldrich (#H8627; purity 98%).

All standard laboratory chemicals and solvents were from Sigma-Aldrich. Cell culture reagents including DMEM (#41965-039), sodium pyruvate (#11360-039), penicillin/streptomycin (#15240-062), and trypsin/EDTA (#15400-054) were from Gibco, Carlsbad, CA, USA, except for FCS (#S181BH from Biowest, Nuaillé, France), PBS (#D8537 from Sigma-Aldrich), and antibiotic-antimycotic solution (#A5955 from Sigma-Aldrich). Cell culture dished and flasks were from TPP, Trasadingen, Switzerland, and used without further surface treatment.

### 4.2. Cell Lines and Their Cultivation

SY5Y human neuroblastoma cells were from LGC Standards, Teddington, UK. Hela human cervical carcinoma cells were from the stocks of the Institute for Pathobiochemistry of the University of Mainz and were authenticated by short tandem repeat (STR) analysis as described [45]. HEK293 immortalized human kidney cells and MCF7 human breast carcinoma cells were from the American Type Culture Collection (ATCC), Manassas, VA, USA. C2C12 mouse myoblast cells were from LGC Standards. HepG2 human hepatocellular carcinoma cells were a kind gift from Dr. Alain Lescure (CNRS, Strasbourg, France).

Cell lines were cultivated at 37 °C in an incubator providing a humidified ambient air atmosphere containing 5% CO_2_. Standard growth medium for all cell types was high-glucose DMEM supplemented with 1 mM pyruvate and 10% heat-inactivated FCS. MCF7 cells were further supplemented with 1× penicillin/streptomycin; SY5Y cells, Hela cells and HEK293 cells received 1× antibiotic-antimycotic solution. During routine culture, the cells were grown in 100 mm dishes and were passaged on reaching approximately 80% confluence (C2C12 cells at 60% confluence).

C2C12 cell differentiation was achieved in 96-well-plates in which the cells had grown to confluence over a course of approximately 3 days. Subsequently, the medium was removed and replaced by serum-free, but otherwise unaltered standard medium. Following 3 days of differentiation, the exhausted medium was exchanged, marking the beginning of the experiment. Hypoxia treatments were performed in a separate incubator that flushed the cultivation chamber with external nitrogen until reaching the desired O_2_ and CO_2_ concentrations. All cells were regularly tested to be negative for contamination with mycoplasma by PCR against the conserved 16S rRNA coding region of the mollicutes using a commercial test kit (Venor GeM Classic from Minerva Biolabs, Berlin, Germany).

### 4.3. Cell Proliferation and Cytotoxicity

The widely employed MTT reduction assay was adopted to a 96-well format in order to quantify cell proliferation and cell survival in response to standardized chemical treatments [46]. Cells were plated at low density in 96-well-plates and cultivated until approximately 25% confluence were reached (within 2–3 days). At this point, parallel plates for the investigational test agents were administered with a minimum of 8 concentrations of each test agent in multiplicates (3–5) for a fixed period of 3 days. All test agents were dissolved as 100× stocks in analytical grade ethanol. Reference plates were supplied with vehicle and analyzed immediately, to yield a control value representing the beginning of the experiment (100% proliferation). The test plates, in turn, were incubated for 3 days under the respective condition, before the same treatment applied to the control plates was identically executed on the test plates. For cell proliferation analysis, the cells were administered with 10 µL MTT solution (5 mg/mL 3-(4,5-dimethylthiazol-2-yl)-2,5-diphenyltetrazolium bromide in ultrapure water) per 100 µL cultivation medium and incubated at 37 °C for a preset time, dependent on the specific cell line (usually 3 h). Subsequently, the cells were lysed with 100 µL solubilization solution (40% dimethylformamide, 10% SDS, pH 4.0 with acetic acid) for 24 h in the dark, after which microscopic homogeneity of the solution was reached. The effectuated cellular MTT reduction was then quantified photometrically at 560 nm with a standard microplate reader. Blanking was done on medium-filled wells in which the cells had been omitted. Interference of the investigational compounds with the assay procedure was also tested and found to be negative at the employed concentrations.

## 5. Conclusions

Thiol-based chain-transfer agents function as prooxidant cytostatics in a variety of cancer cell lines in vitro. They show similar molar potency as different clinically established anti-cancer drugs, but they may be of lower systemic toxicity due to their mode of action requiring activation by endogenous free radicals. Chain-transfer agents target tumor cells independently of the classic mechanisms (rapid cell division, DNA synthesis, and tumor antigens), but rather exploit the higher levels of initiator free radicals found in many tumor cells. In modern combination therapy, they might thus add an extra level of specificity to standard triple-therapeutic regimens. They might also find their role in the adjuvant amplification of standard radiotherapy, which essentially acts by inducing initiator radicals in the first place.

## 6. Patents

The University Medical Center of the Johannes Gutenberg University, Mainz, Germany, has filed a patent pertaining to the use of chain-transfer agents as medicinal drugs (PCT Int. Appl. (2021), 44 pp., WO 2021/105435).

## Figures and Tables

**Table 1:** Selected properties of the thiols and thioethers investigated in this work.

Compound	Abbreviation	Purity	Lipophilicity (logP)
Octane-1-thiol	8SH	98.5%	3.47
Decane-1-thiol	10SH	99%	4.30
Dodecane-1-thiol	12SH	98%	5.14
Tetradecane-1-thiol	14SH	98%	5.97
Hexadecane-1-thiol	16SH	97%	6.80
Octadecane-1-thiol	18SH	98%	7.64
1-Methylsulfanyldodecane	12SMe	97%	5.39

**Table 2:** Half-maximal effective concentrations (µM) of the chain-transfer agents and the control compounds investigated in this work.

Compound	SY5Y	SY5Y, 1% O_2_	Hela	HEK293	MCF7	C2C12	C2C12, Differ.	HepG2
8SH	>100	>100	-	-	>100	-	-	-
10SH	5 ± 1	4 ± 2	5 ± 3	20 ± 8	9 ± 1	5 ± 1	60 ± 40	>100
12SH	4 ± 1	4 ± 1	1 ± 0.2	4 ± 1	9 ± 1	0.7 ± 0.2	>100	>100
14SH	2 ± 0.5	2 ± 0.5	2 ± 0.5	9 ± 5	6 ± 2	3 ± 1	10 ± 2	90 ± 40
16SH	2 ± 0.5	4 ± 1	2 ± 1	-	-	-	-	-
18SH	0.8 ± 0.5	6 ± 4	1 ± 0.2	4 ± 3	2 ± 1	30 ± 8	>100	>100
12SMe	>100	>100	>100	>100	40 ± 10	>100	>100	>100
Dox	0.05 ± 0.01	-	<0.01	-	-	-	-	-
Act	2 ± 1	-	0.3 ± 0.1	-	-	-	-	-
FU	5 ± 1	-	0.8 ± 0.2	-	-	-	-	-
HU	80 ± 20	-	70 ± 20	-	-	-	-	-

## Data Availability

The data presented in this study are available on request from the corresponding author.

## Sample Availability

All chemicals and cell lines employed in this work are available from commercial sources.

## References

[1] Siegel R.L., Miller K.D., Jemal A. (2019). Cancer statistics, 2019. CA Cancer J. Clin..

[2] Dolgin E. (2018). Bringing down the cost of cancer treatment. Nature.

[3] Sessa C., Gianni L., Garassino M., van Halteren H. (2012). ESMO Handbook of Clinical Pharmacology of Anticancer Agents.

[4] Trachootham D., Alexandre J., Huang P. (2009). Targeting cancer cells by ROS-mediated mechanisms: A radical therapeutic approach?. Nat. Rev. Drug Discov..

[5] Wallace D.C. (2012). Mitochondria and cancer. Nat. Rev. Cancer.

[6] Gorrini C., Harris I.S., Mak T.W. (2013). Modulation of oxidative stress as an anticancer strategy. Nat. Rev. Drug Discov..

[7] Sosa V., Moliné T., Somoza R., Paciucci R., Kondoh H., LLeonart M.E. (2013). Oxidative stress and cancer: An overview. Ageing Res. Rev..

[8] Doskey C.M., Buranasudja V., Wagner B.A., Wilkes J.G., Du J., Cullen J.J., Buettner G.R. (2016). Tumor cells have decreased ability to metabolize H2O2: Implications for pharmacological ascorbate in cancer therapy. Redox Biol..

[9] Szatrowski T.P., Nathan C.F. (1991). Production of large amounts of hydrogen peroxide by human tumor cells. Cancer Res..

[10] Kumar B., Koul S., Khandrika L., Meacham R.B., Koul H.K. (2008). Oxidative stress is inherent in prostate cancer cells and is required for aggressive phenotype. Cancer Res..

[11] Moss R.W. (2007). Do antioxidants interfere with radiation therapy for cancer?. Integr. Cancer Ther..

[12] Barker H.E., Paget J.T., Khan A.A., Harrington K.J. (2015). The tumour microenvironment after radiotherapy: Mechanisms of resistance and recurrence. Nat. Rev. Cancer.

[13] Dolmans D.E., Fukumura D., Jain R.K. (2003). Photodynamic therapy for cancer. Nat. Rev. Cancer.

[14] Toler S.M., Noe D., Sharma A. (2006). Selective enhancement of cellular oxidative stress by chloroquine: Implications for the treatment of glioblastoma multiforme. Neurosurg. Focus.

[15] Cui Q., Wen S., Huang P. (2017). Targeting cancer cell mitochondria as a therapeutic approach: Recent updates. Future Med. Chem..

[16] Kubli S.P., Bassi C., Roux C., Wakeham A., Göbl C., Zhou W., Jafari S.M., Snow B., Jones L., Palomero L. (2019). AhR controls redox homeostasis and shapes the tumor microenvironment in BRCA1-associated breast cancer. Proc. Natl. Acad. Sci. USA.

[17] Kunath S., Schindeldecker M., De Giacomo A., Meyer T., Sohre S., Hajieva P., von Schacky C., Urban J., Moosmann B. (2020). Prooxidative chain transfer activity by thiol groups in biological systems. Redox Biol..

[18] Gridnev A.A., Ittel S.D. (2001). Catalytic chain transfer in free-radical polymerizations. Chem. Rev..

[19] Dietrich B.K., Pryor W.A., Wu S.J. (1988). Chain transfer constants of mercaptans in the emulsion polymerization of styrene. J. Appl. Polym. Sci..

[20] Moad G., Rizzardo E., Thang S.H. (2012). Living Radical Polymerization by the RAFT Process—A Third Update. Aust. J. Chem..

[21] Nicolas J., Guillaneuf Y., Lefay C., Bertin D., Gigmes D., Charleux B. (2013). Nitroxide-mediated polymerization. Prog. Polym. Sci..

[22] Odian G., Odian G. (2004). Radical chain polymerization. Principles of Polymerization.

[23] Moosmann B. (2011). Respiratory chain cysteine and methionine usage indicate a causal role for thiyl radicals in aging. Exp. Gerontol..

[24] Moosmann B., Schindeldecker M., Hajieva P. (2020). Cysteine, glutathione and a new genetic code: Biochemical adaptations of the primordial cells that spread into open water and survived biospheric oxygenation. Biol. Chem..

[25] Fuhrmeister J., Tews M., Kromer A., Moosmann B. (2012). Prooxidative toxicity and selenoprotein suppression by cerivastatin in muscle cells. Toxicol. Lett..

[26] Hughes V.S., Wiggins J.M., Siemann D.W. (2019). Tumor oxygenation and cancer therapy-then and now. Br. J. Radiol..

[27] Brizel D.M., Scully S.P., Harrelson J.M., Layfield L.J., Bean J.M., Prosnitz L.R., Dewhirst M.W. (1996). Tumor oxygenation predicts for the likelihood of distant metastases in human soft tissue sarcoma. Cancer Res..

[28] Monteiro A.R., Hill R., Pilkington G.J., Madureira P.A. (2017). The role of hypoxia in glioblastoma invasion. Cells.

[29] Lohitesh K., Chowdhury R., Mukherjee S. (2018). Resistance a major hindrance to chemotherapy in hepatocellular carcinoma: An insight. Cancer Cell Int..

[30] Pacifici G.M., Santerini S., Giuliani L., Rane A. (1991). Thiol methyltransferase in humans: Development and tissue distribution. Dev. Pharmacol. Ther..

[31] Lipton S.A. (2007). Pathologically activated therapeutics for neuroprotection. Nat. Rev. Neurosci..

[32] Negre-Salvayre A., Auge N., Ayala V., Basaga H., Boada J., Brenke R., Chapple S., Cohen G., Feher J., Grune T. (2010). Pathological aspects of lipid peroxidation. Free Radic. Res..

[33] Tudek B., Zdżalik-Bielecka D., Tudek A., Kosicki K., Fabisiewicz A., Speina E. (2017). Lipid peroxidation in face of DNA damage, DNA repair and other cellular processes. Free Radic. Biol. Med..

[34] Kunath S., Moosmann B. (2020). What is the rate-limiting step towards aging? Chemical reaction kinetics might reconcile contradictory observations in experimental aging research. Geroscience.

[35] Moosmann B. (2021). Flux control in the aging cascade. Aging.

[36] Moosmann B., Behl C. (2008). Mitochondrially encoded cysteine predicts animal lifespan. Aging Cell.

[37] Pradas I., Huynh K., Cabré R., Ayala V., Meikle P.J., Jové M., Pamplona R. (2018). Lipidomics reveals a tissue-specific fingerprint. Front. Physiol..

[38] Jové M., Mota-Martorell N., Pradas I., Galo-Licona J.D., Martín-Gari M., Obis È., Sol J., Pamplona R. (2020). The lipidome fingerprint of longevity. Molecules.

[39] Bartoli G.M., Bartoli S., Galeotti T., Bertoli E. (1980). Superoxide dismutase content and microsomal lipid composition of tumours with different growth rates. Biochim. Biophys. Acta.

[40] Peck B., Schug Z.T., Zhang Q., Dankworth B., Jones D.T., Smethurst E., Patel R., Mason S., Jiang M., Saunders R. (2016). Inhibition of fatty acid desaturation is detrimental to cancer cell survival in metabolically compromised environments. Cancer Metab..

[41] Szlasa W., Zendran I., Zalesińska A., Tarek M., Kulbacka J. (2020). Lipid composition of the cancer cell membrane. J. Bioenerg. Biomembr..

[42] n-Dodecyl Mercaptan (2019). Safety Data Sheet, Version 4.14.

[43] Fluorouracil Injection (2012). Safety Data Sheet, Version 1.1.

[44] Actinomycin D. (2021). Safety Data Sheet, Version 7.0.

[45] Bekbulat F., Schmitt D., Feldmann A., Huesmann H., Eimer S., Juretschke T., Beli P., Behl C., Kern A. (2020). RAB18 loss interferes with lipid droplet catabolism and provokes autophagy network adaptations. J. Mol. Biol..

[46] Hajieva P., Bayatti N., Granold M., Behl C., Moosmann B. (2015). Membrane protein oxidation determines neuronal degeneration. J. Neurochem..

